# Trends in teicoplanin loading dose implementation from 2010 to 2019 and evaluation of safety and efficacy factors: a retrospective cohort study based on a Japanese administrative claims database

**DOI:** 10.1186/s40780-023-00304-y

**Published:** 2023-11-01

**Authors:** Ryota Goto, Yuichi Muraki, Ryo Inose, Moeno Ichii, Keisuke Sawada, Kanako Mizuno, Ryuji Koizumi, Shinya Tsuzuki, Masahiro Ishikane, Norio Ohmagari

**Affiliations:** 1 Department of Clinical Pharmacoepidemiology, Kyoto Pharmaceutical University, 5 Misasagi Nakauchi-Cho, Yamashina-Ku, Kyoto, 607-8414 Japan; 2 Department of Pharmacy, Federation of National Public Service Personnel Mutual Aid Associations, Hirakata Kohsai Hospital, 1-2-1 Fujisaka Higashimachi, Osaka, 573-0153 Japan; 3 AMR Clinical Reference Center, Disease Control and Prevention Center, National Center for Global Health and Medicine, 1-21-1 Toyama Shinjuku-Ku, Tokyo, 162-8655 Japan

**Keywords:** Teicoplanin, Database, Loading dose, Liver injury, Mortality, Methicillin-resistant *Staphylococcus aureus*, Japan

## Abstract

**Background:**

The loading dose of teicoplanin (TEIC) is recommended for implementation. However, there is significant discrepancy between the dose settings in the package insert and, in the guidelines, and the actual status of loading doses in Japan is unclear. Furthermore, TEIC causes liver injury as side effect. Although the risk of developing liver injury has not been reported to be increased following a loading dose based on the guidelines, there is a lack of reports in large populations. Therefore, we evaluated the trend in the loading dose and factors affecting the efficacy and safety of TEIC administration.

**Methods:**

A Japanese administrative claims database was used in this study. Trends in loading doses were evaluated in target populations administered TEIC between 2010 and 2019. Patient characteristics were adjusted by propensity score matching based on the guideline group (total dose of 3 days > 1,600 mg) and non-guideline group (≤ 1,600 mg) of the loading dose. Finally, univariable and multivariable conditional logistic regression analysis was performed to evaluate factors affecting 30-day mortality and liver injury.

**Results:**

A total of 10,030 patients were selected based on these criteria. The proportion of loading doses based on the recommended guidelines showed an increase over time, regardless of the implementation of therapeutic drug monitoring (TDM), but especially so in cases where TDM was implemented, the loading doses were administered in accordance with the recommendations of the guidelines. Conditional logistic regression analysis showed a relationship between drug management and guidance fees (odds ratio [OR]: 0.45, 95% confidence interval [CI]: 0.36‒0.55), a reimbursement indicating pharmacist intervention, and a reduction in 30-day mortality. In addition, loading doses based on the recommended guidelines had no influence on liver injury, and other factors were not significantly associated with increased incidence of liver injury.

**Conclusion:**

Thus, this study implies the benefits of pharmacological management as indicated by drug management and guidance fee and supports the implementation of loading doses based on the guideline on TEIC administration.

**Supplementary Information:**

The online version contains supplementary material available at 10.1186/s40780-023-00304-y.

## Background

In recent years, antimicrobial-resistant bacteria has become a global problem [1]. In 2019, the antimicrobial resistance collaborator estimated that there were approximately 1.27 million deaths worldwide caused by antimicrobial-resistant bacteria [2]. In addition, over 100,000 of these deaths were caused by Methicillin-resistant *Staphylococcus aureus* (MRSA), a representative antimicrobial-resistant bacterium, and the need for countermeasures is extremely high [2]. Vancomycin (VCM) is a standard drug used worldwide for the treatment of infections caused by MRSA [3, 4]. However, VCM is known to cause acute kidney injury (AKI) as a side effect [5]. Therefore, teicoplanin (TEIC) is recommended as an alternative to VCM in practical guidelines for the management and treatment of infections caused by MRSA in Japan [6].

Similar to VCM, TEIC is a glycopeptide anti-MRSA agent [7]. In a meta-analysis, TEIC was reported to have a lower risk of side effects such as nephrotoxicity and red man syndrome than VCM [8]. In contrast, liver injury is mentioned as a side effect in the TEIC (Targocid®) package insert [9]. In addition, TEIC has a long half-life, and the dose setting is based on the implementation of a loading dose to increase the blood concentration at an early stage. Therefore, the implementation of therapeutic drug monitoring (TDM) is recommended for the administration of TEIC in terms of efficacy and safety [10]. However, there is a significant discrepancy between the loading dose settings in the package inserts [9] and the guidelines [10]; and the actual loading dose status in Japan is unclear.

It is assumed that trough levels of 15–30 µg/mL can be reached with higher loading doses as recommended in the guidelines. In this case, meta-analyses reported enhanced efficacy without increasing the risk of side effects in meta-analyses [11]. However, most of these reports were obtained from the same institution. Therefore, multi-institutional studies have not been adequately conducted. In addition, the factors influencing the safety and efficacy of TEIC administration remain unclear.

In recent years, studies have increasingly used real-world data (RWD), containing patient health status and/or the routinely delivered healthcare, collected from a variety of sources [12]. Among RWD, administrative claims databases have accumulated information on the diagnosis and treatment of patients at many facilities and are used to evaluate the risk of side effects and treatment effects [13, 14]. Therefore, we examined the implementation of loading doses of TEIC and the factors affecting the safety and efficacy of TEIC based on the administrative claims database.

## Methods

### Data source

This study used an administrative claims database collected from hospitals marketed by Medical Data Vision Co., Ltd. [15]. The database has over 40 million patient records and covers approximately 26% of all hospitals in Japan that have adopted a diagnosis procedure combination system. From this data, it is possible to obtain a variety of information related to patient treatments, such as diagnoses, prescription drugs, surgeries, and procedures. The data used were anonymized so that individual patients could not be identified and informed consent was not obtained.

### Selection

The study population consisted of patients administered TEIC between 2010 and 2019. Patients aged 15 years or older who were administered TEIC for at least three consecutive days were identified from this population, and the date of the first TEIC administration was defined as the index date. We restricted our sample to patients for whom some data existed up to the month preceding the index date. Then, patients who had received ursodeoxycholic acid (UDCA) and/or glycyrrhizin (GL) as of the month preceding the index date were excluded to ensure that patients with suspected pre-existing liver injuries were excluded. Because TDM was defined as a claim for treatment and management fees for specific drugs, it excluded patients who had been treated with a TDM drug, except for TEIC, within seven days of the index date (Additional file 1: Table S1). Finally, patients with missing data were excluded, and those that met the inclusion criteria for the study were selected.

### Definition

Validation studies on positive predictive value of liver injury definition using the International Classification of Diseases, 10th edition (ICD-10) codes have not been conducted in Japan. Therefore, in this study, liver injury was defined as a diagnosis indicated by ICD-10 codes (K711/K719 [toxic drug-induced liver injury], K720 [acute and subacute liver injury], K769 [unspecified liver injury]) and treatment indicated by the administration of UDCA and/or GL recorded in the same month. Additionally, all suspected diagnoses were excluded. The treatment was defined as another treatment if there was an interval of 7 days between teicoplanin doses, and the treatment duration was defined as the period from the index date to the final administration date of the first treatment.

Teicoplanin was described in the package insert as 400 mg or 800 mg in two divided doses on the first day and 200 mg or 400 mg once daily thereafter [9]. In addition, TDM guidelines recommend doses higher than the package insert [10]. Therefore, the loading dose of TEIC was calculated for a total dose of 3 days from the index date. Loading doses were classified into the following three categories: 800–1,600 mg (the package insert dose),  > 1,600 mg (guideline dose), and  < 800 mg.

TDM was defined as reimbursement claims of treatment and management fees for specific drugs, according to previous reports [16, 17]. Similarly, pharmacist management for individual patients, placement of pharmacists on hospital wards, and establishment of infection control teams in healthcare facilities were defined by drug management and guidance fees, inpatient pharmaceutical service premiums, and infection prevention and control premiums, respectively [17].

Furthermore, no reports or guidelines clearly define drugs with potential risk of liver injury. Therefore, in this study, drugs that have already been reported to cause liver injury were classified according to their effects based on the serious disease manual, which is regularly updated by the Ministry of Health, Labor and Welfare (MHLW) [18]. Among these, drugs with a high frequency of risk occurrence for liver injury were classified into the following six categories: anti-infection drugs, antipyretic analgesics and anti-inflammatory drugs, anticancer drugs, gastrointestinal drugs, psychiatric or neurological drugs, and metabolic disease drugs. These drugs were defined as concomitant medications if their administration overlapped with the treatment period of TEIC.

### Data collection

Data on concomitant drugs at risk of liver injury were extracted from among oral and injectable drugs containing the relevant ingredients [18]. In addition, as in previous reports [19], the site of infection was defined using the Japanese disease code that is uniquely assigned to each disease. Details are provided in the supplementary files (Additional file 2: Table S2, Additional file 3: Table S3).

Diagnostic information for the month of admission was used to calculate Charlson Comorbidity Index (CCI). Scores were calculated using a program that can be introduced into the Stata software version 17.0 (Stata Corp., College Station, TX, USA).

### Statistical analysis

The implementation of loading doses during TEIC administration was surveyed over time and compared after classifying the implementation of TDM. In the target population, propensity score matching was performed based on loading doses according to the guideline doses (guideline group) or below the package insert dose (non-guideline group). The nearest neighbor within caliper without replacement was used, and patient background was adjusted. The propensity score was calculated using logistic regression, and 1:1 matching was performed using a caliper of 0.2. Clinical variables included information that might influence the loading dose and that could be collected clinically before the start of TEIC treatment. In this study, bed size, age, CCI, weight, reimbursement, site of infection, and clinical department were selected. The balance between the two groups was evaluated using the Mann–Whitney *U* test for continuous variables and the chi-square test for categorical variables in the before propensity score matching population, and standardized mean difference (SMD) in the matched population. SMD was considered balanced if it was  < 0.1. In addition, risk factors for 30-day mortality and liver injury in patients administered TEIC were evaluated using univariable and multivariable conditional logistic regression analysis.

We used Stata software and EZR software (Saitama Medical Center, Jichi Medical University, Saitama, Japan) [20] for statistical analysis, with a significance level set at *p* < 0.05.

## Results

### Patient selection

**Fig. 1:**
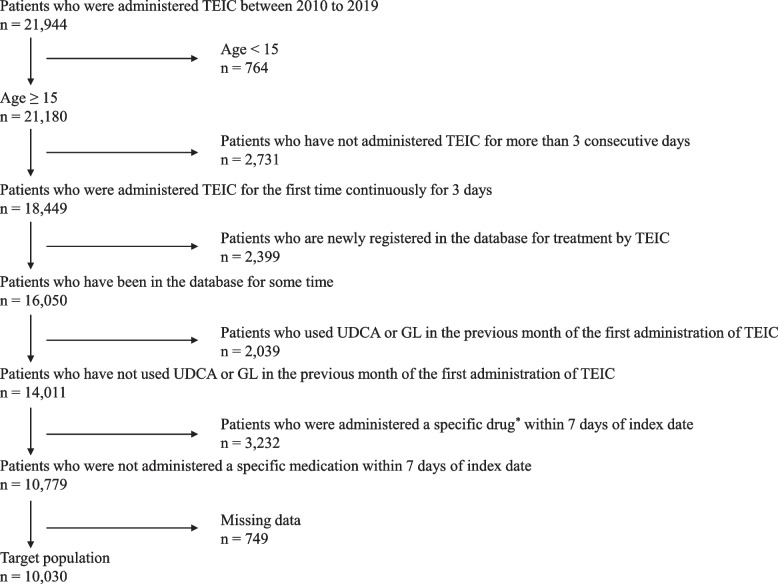
Patient selection. *TEIC* Teicoplanin, *UDCA* ursodeoxycholic acid, *GL* glycyrrhizin. ^*^A specific drug refers to a drug for which a claim can be made for treatment and management fee for specific drugs, and based on the drug whose blood levels are assumed to be clinically measured (Additional file 1)

The patient selection flowchart is as shown in Fig. 1. The study population included 21,944 patients administered TEIC between 2010 and 2019. According to the patient selection criteria, 10,030 eligible patients met the inclusion criteria for age, administration period, enrollment in database, drug history, and concomitant drug use.

### Implementation of loading doses

**Fig. 2:**
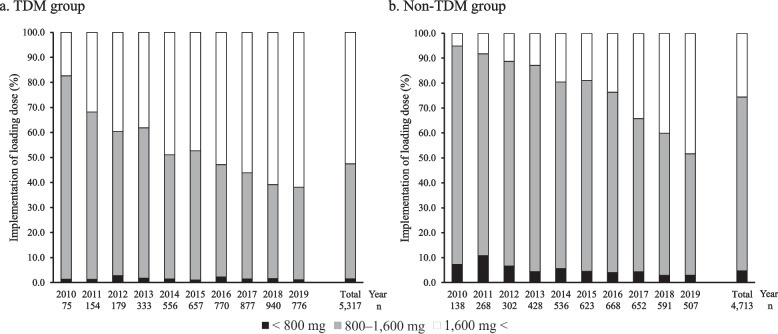
Trend in the implementation of loading dose by TDM from 2010 to 2019. *TDM* therapeutic drug monitoring

Trends in the implementation of loading doses in TEIC over time are shown in Fig. 2. From 2010 to 2019, the implementation of loading doses based on the guidelines increased, regardless of the implementation of TDM. As shown in Fig. 2, over the whole period, the proportion of patients who received loading doses based on guideline was higher with TDM (52.5%) compared to without TDM (25.5%). In addition, the proportion of patients administered less than the package insert dose setting was higher in those without TDM (4.73%) than in those with TDM (1.56%).

### Evaluation of factors related to efficacy and safety of TEIC

As Table 1 shows, the patient characteristics were partially uneven between the two groups: guideline group (> 1,600 mg) and non-guideline group (≤ 1,600 mg). After propensity score matching, 3,309 patients were included in each group adjusted for patient background.

**Table 1:** Patient characteristics and propensity score matching based on loading dose

	Before propensity score matching			After propensity score matching		
	1,600 mg < (3,998)	≤ 1,600 mg (6,032)	*p* value	1,600 mg < (3,309)	≤ 1,600 mg (3,309)	SMD
**Number of beds**^a^						
≤ 199	181 (4.5)	596 (9.9)		180 (5.4)	184 (5.6)	
200–499	1,834 (45.9)	3,586 (59.4)	<0.001	1,727 (52.2)	1,731 (52.3)	0.007
≥ 500	1,983 (49.6)	1,850 (30.7)		1,402 (42.4)	1,394 (42.1)	
**CCI** ^b^	2 [1‒4]	2 [1‒4]	0.32	2 [1‒4]	2 [1‒4]	0.022
**Age (years)** ^b^	75 [65‒83]	76 [67‒84]	< 0.001	76 [66‒84]	75 [66‒83]	0.016
**Weight** ^b^	54.8 [46.6‒63.8]	51.6 [43.6‒60.1]	< 0.001	53.2 [45.4‒62.5]	53 [45‒62]	0.016
**Reimbursement**^a^						
Infection prevention and control premium	3,165 (79.2)	4,504 (74.7)	< 0.001	2,601 (78.6)	2,585 (78.1)	0.012
Inpatient pharmaceutical service premium	2,438 (61.0)	2,112 (35.0)	< 0.001	1,762 (53.2)	1,802 (54.5)	0.024
**Site of infection**^a^						
Respiratory infection	1,392 (34.8)	2,569 (42.6)	< 0.001	1,278 (38.6)	1,246 (37.7)	0.020
Bacteremia/sepsis	1,302 (32.6)	2,030 (33.7)	0.26	1,110 (33.5)	1,092 (33.0)	0.012
Urinary tract infections	333 (8.3)	579 (9.6)	0.03	294 (8.9)	294 (8.9)	< 0.001
Intra-abdominal infections	276 (6.9)	318 (5.3)	0.001	194 (5.9)	201 (6.1)	0.009
Skin and soft tissue infection	348 (8.7)	391 (6.5)	< 0.001	274 (8.3)	267 (8.1)	0.008
Bone and joint infections	337 (8.4)	336 (5.6)	< 0.001	259 (7.8)	241 (7.3)	0.021
Central nervous system infections	45 (1.1)	49 (0.8)	0.11	32 (1.0)	34 (1.0)	0.006
Infective endocarditis	40 (1.0)	37 (0.6)	0.03	32 (1.0)	29 (0.9)	0.009
**Clinical department**^a^						
Internal medicine	827 (20.7)	2,037 (33.8)		806 (24.4)	839 (25.4)	
Hematology	385 (9.6)	912 (15.1)		354 (10.7)	372 (11.2)	
Surgery	456 (11.4)	695 (11.5)	< 0.001	405 (12.2)	380 (11.5)	0.036
Orthopedic surgery	410 (10.3)	449 (7.4)		316 (9.5)	316 (9.5)	
Others	1920 (48.0)	1,939 (32.1)		1,428 (43.2)	1,402 (42.4)	

*CCI* Charlson comorbidity index, *SMD* standardized mean difference

^a^Data are expressed n (%), and Chi-square test was performed for the significant difference test

^b^Data are expressed median [interquartile rate], and Mann–Whitney *U* test was performed for the significant difference test

Table 2 shows the results of the conditional logistic regression analysis of 30-day mortality. A total of 1,214 (18.3%) patients died within 30 days of TEIC administration. The implementation of guideline-based loading dose was significantly related to an increase of 30-day mortality (odds ratio [OR]: 1.20, 95% confidence interval [CI]: 1.04–1.39). Drug management and guidance fees (OR: 0.45, 95% CI: 0.36‒0.55) were related to reduced 30-day mortality.

**Table 2:** Clinical variables related to 30-day mortality in the administration of teicoplanin

	Univariable			Multivariable		
	OR	95% CI	*p* value	OR	95% CI	*p* value
**Patient information**						
Sex [female vs. male]	1.26	1.05‒1.51	0.014	1.29	1.06‒1.57	0.013
**Treatment details**						
Treatment duration (day)	0.94	0.93‒0.95	< 0.001	0.94	0.93‒0.95	< 0.001
Total dose for 3 days [≤ 1,600 mg vs. 1,600 <]	1.14	1.01‒1.30	0.032	1.20	1.04‒1.39	0.013
**Reimbursement**						
Treatment and management fee for specific drugs	1.04	0.88‒1.24	0.63	1.12	0.91‒1.37	0.28
Drug management and guidance fee	0.44	0.37‒0.53	< 0.001	0.45	0.36‒0.55	< 0.001

*CCI* Charlson comorbidity index, *OR* odds ratio, *95% CI* 95% confidence interval

Table 3 shows the results of the conditional logistic regression analysis of the incidence of liver injury. A total of 124 (1.87%) patients experienced liver injury after the TEIC administration. There was no significant influence on the total dose for 3 days. In addition, no other factor was identified that significantly increased liver injury.

**Table 3:** Clinical variables related to liver injury in the administration of teicoplanin

	Univariable			Multivariable		
	OR	95% CI	*p* value	OR	95% CI	*p* value
**Treatment details**						
Treatment duration (day)	0.98	0.95‒1.01	0.16	0.97	0.94‒1.00	0.078
Total dose for 3 days [≤ 1,600 mg vs. 1,600 <]	1.11	0.77‒1.60	0.58	0.99	0.63‒1.55	0.96
**Reimbursement**						
Treatment and management fee for specific drugs	1.07	0.64‒1.77	0.80	1.11	0.59‒2.09	0.74
Drug management and guidance fee	1.36	0.79‒2.36	0.27	1.48	0.78‒2.82	0.24
**Concomitant drug**						
Anti-infection drugs	0.52	0.28‒0.94	0.032	0.43	0.22‒0.82	0.011
Antipyretic analgesics and anti-inflammatory drugs	1.42	0.86‒2.35	0.17	1.61	0.90‒2.88	0.11
Anticancer drugs	1.33	0.30‒5.96	0.71	1.58	0.29‒8.69	0.60
Gastrointestinal drugs	1.06	0.55‒2.05	0.87	1.12	0.53‒2.37	0.77
Psychiatric or neurological drugs	0.75	0.43‒1.32	0.32	0.61	0.32‒1.14	0.12
Metabolic diseases drugs	1.15	0.68‒1.95	0.59	1.41	0.79‒2.52	0.24

*CCI* Charlson comorbidity index, *OR* odds ratio, *95% CI* 95% confidence interval

## Discussion

TEIC is an important drug, because it is an alternative to VCM. In this study, we evaluated the trend in the loading dose of TEICs in Japan and the factors influencing 30-day mortality and the incidence of liver injury in patients administered TEICs.

In this study, we observed an increase in the loading dose over time in accordance with the guidelines rather than the package inserts. In addition, the TDM group was more compliant with the guideline recommendations than the non-TDM group. The guidelines in Japan were first published in 2012 and subsequently revised in 2016. The guidelines include content on TDM and loading dose setting, and clinical and evidence-based treatments are becoming better known. Furthermore, the guidelines were revised in 2022, but with continued recommendations of higher loading doses [21], it is expected that the implementation of loading doses will increase further in the future. Therefore, it may be necessary to revise the package inserts.

However, in cases where TDM was not performed, compared to cases where TDM was performed, there were some inappropriate uses in which less than the dose setting on the package insert was administered. It has been reported that 14 days of repeated administration is required to achieve a steady state without loading doses [22]. Therefore, it is necessary to survey the objectives of administration in more detail for patients treated without TDM or appropriate loading doses in future studies.

The loading dose based on the guidelines was one of the factors that increased the 30-day mortality. The trough levels should be set at more than 20 µg/mL in severe cases and in complicated infections [10]. It is possible that higher loading doses of TEIC were administered to patients with a higher risk of death. Moreover, in terms of reimbursement, claiming drug management and guidance fee was related to a decrease in the 30-day mortality. As we have shown in patients administered VCM [19], the results suggested that it may be important to enhance the effectiveness of treatment promoting pharmacological management of individual patients by the pharmacist.

The guideline-based loading dose did not affect the incidence of liver injury. Trough levels of 15–30 µg/mL are reported to be achieved in dose setting with loading doses based on the guideline [23]. The trough levels of 15–30 μg/mL have been reported in single-center studies to have no significant difference in the incidence of liver injury compared to trough levels of less than 15 μg/mL [11]. In this study using RWD, a similar trend was observed, and the results may support the evidence of safety at higher dose of TEIC. No other factor was identified as significantly associated with an increase in the incidence of liver injury. In contrast, anti-infection drugs were significantly associated with a decrease in the incidence of liver injury. The basis for this result is unknown and requires further investigation.

This study has several limitations. First, there are limitations related to the use of databases and definition. In this study, we used hospital-based administrative claims database. Therefore, due to shortcomings in patient traceability, it is difficult to assess information on diagnoses and prescriptions at other hospitals, and this may have affected the results. Also, we defined liver injury as a combination of information on diagnosis using ICD-10 codes and information on medications such as UDCA and GL. However, it is not clear whether these codes or drugs are directly related to teicoplanin-induced liver injury. Besides, the actual occurrence of liver injury may be underestimated because this definition is limited to severe cases requiring drug treatment. Actually, the incidence of liver injury in this study was low compared to 3.3–5.5% reported previously [11]. Furthermore, because the order of diagnosis and prescription could not be clarified, the impact of the initiation of concomitant drugs after the occurrence of liver injury could not be assessed. Second, there were some confounding issues that were difficult to investigate, such as the actual state of liver function and trough levels of TEIC. Third, the definition of loading dose was defined as the total dose of 3 days. Therefore, it may include patients treated with maintenance doses rather than loading doses, e.g., 600 mg once daily. However, from data confirmation, the observed number of affected patients was only 17 out of 10,030 (0.169%), and the impact is considered slight. Nevertheless, this study could be useful information in the treatment with TEIC, a valuable alternative to VCM.

## Conclusions

It was clear that the loading dose of TEIC based on the guidelines rather than that of the package inserts has increased over time. In addition, it was found that the implementation of pharmacological management may enhance the effectiveness of the treatment in the administration of TEIC. Moreover, it was shown that higher loading doses based on the guidelines may not be a risk factor for liver injury based on the administrative claim database. In the future, applying this approach enabled us to examine recommendations that have not been adequately validated in large populations regarding the loading dose, safety, or efficacy.

### Supplementary Information

**Additional file 1: Table S1.** Drugs corresponding to specific drugs.

**Additional file 2: Table S2.** Concomitant drugs at risk of liver injury.

**Additional file 3: Table S3.** Codes for classification of site of infection.

## Data Availability

We purchased and used the data from Medical Data Vision. Therefore, these data are not publicly available. If other researchers wish to use the data, they need to purchase it, along with the authors.

## Abbreviations

AKI: Acute kidney injury
CCI: Charlson comorbidity index
GL: Glycyrrhizin
ICD-10: International classification of diseases 10th revision
MHLW: Ministry of Health, Labor and Welfare
MRSA: Methicillin-resistant *Staphylococcus aureus*
RWD: Real-world data
SMD: Standardized mean difference
TDM: Therapeutic drug monitoring
TEIC: Teicoplanin
UDCA: Ursodeoxycholic acid
VCM: Vancomycin
