# Supplementary material for: Trends in teicoplanin loading dose implementation from 2010 to 2019 and evaluation of safety and efficacy factors: a retrospective cohort study based on a Japanese administrative claims database
Source: J Pharm Health Care Sci. 2023 Nov 1;9:35. doi: 10.1186/s40780-023-00304-y (PMC10619217; doi:10.1186/s40780-023-00304-y)
Supplement: Supplementary file 1 — Additional file 1: Table S1. Drugs corresponding to specific drugs. [file 40780_2023_304_MOESM1_ESM.docx]

Additional file 1: Table S1. Drugs corresponding to specific drugs

| Drug name |  | |  |
| --- | --- | --- | --- |
| **Antiarrhythmic agents** |  | |  |
| Amiodarone hydrochloride | Aprindine hydrochloride | | Bepridil hydrochloride hydrate |
| Cibenzoline succinate | Disopyramide | | Disopyramide phosphate |
| Flecainide acetate | Mexiletine hydrochloride | | Pilsicainide hydrochloride hydrate |
| Pirmenol hydrochloride hydrate | Procainamide | | Propafenone hydrochloride |
| Quinidine sulfate hydrate | Sotalol hydrochloride | |  |
| **Antiepileptic drugs** |  | |  |
| Carbamazepine | Fosphenytoin | | Phenobarbital |
| Phenobarbital sodium | Phenytoin | | Phenytoin sodium |
| Phenytoin-phenobarbital combination | |  | Sodium valproate |
| **Aminoglycoside antibiotic** |  | |  |
| Amikacin sulfate | Arbekacin sulfate | | Gentamicin sulfate |
| Tobramycin |  | |  |
| **Digitalis preparation** |  | |  |
| Digoxin | Methyldigoxin | |  |
| **Glycopeptide antibiotic** |  | |  |
| vancomycin |  | |  |
| **Imatinib** |  | |  |
| Imatinib mesylate |  | |  |
| **Immunosuppressive drug** |  | |  |
| Ciclosporin | Everolimus | | Mycophenolate mofetil |
| Tacrolimus hydrate |  | |  |
| **Lithium preparation** |  | |  |
| Lithium carbonate |  | |  |
| **Sirolimus preparation** |  | |  |
| Sirolimus |  | |  |
| **Sunitinib** |  | |  |
| Sunitinib malate |  | |  |
| **Theophylline preparation** |  | |  |
| Theophylline |  | |  |
| **Triazole antifungal agent** |  | |  |
| Voriconazole |  | |  |
