# Supplementary material for: Trends in teicoplanin loading dose implementation from 2010 to 2019 and evaluation of safety and efficacy factors: a retrospective cohort study based on a Japanese administrative claims database
Source: J Pharm Health Care Sci. 2023 Nov 1;9:35. doi: 10.1186/s40780-023-00304-y (PMC10619217; doi:10.1186/s40780-023-00304-y)
Supplement: Supplementary file 2 — Additional file 2: Table S2. Concomitant drugs at risk of liver injury. [file 40780_2023_304_MOESM2_ESM.docx]

Additional file 2: Table S2. Concomitant drugs at risk of liver injury

| Drug name |  |  |
| --- | --- | --- |
| **Anti-infection drugs** |  |  |
| Clarithromycin | Levofloxacin | Cefazolin Sodium |
| Cefcapene Pivoxil | Isoniazid | Daclatasvir |
| Asunaprevir | Azithromycin | Cefditoren Pivoxil |
| Garenoxacin | Sulfamethoxazole, Trimethoprim | Terbinafine |
| Amoxicillin | Cefpodoxime Proxetil | Cefepime |
| Sulbactam Sodium, Ampicillin Sodium | Tazobactam, Piperacillin | Meropenem |
| Faropenem | Clindamycin Phosphate | Ceftriaxone Sodium |
| Cefalexin | Tosufloxacin |  |
| **Antipyretic analgesics and anti-inflammatory drugs** |  |  |
| Loxoprofen | Acetaminophen | Tramadol, Acetaminophen |
| Diclofenac | Meloxicam | Ibuprofen |
| Mefenamic Acid | lornoxicam | Naproxen |
| Neurotropin | Isopropylantipyrine, Acetaminophen, Allylisopropylacetylurea, Anhydrous Caffeine | Salicylamide, Acetaminophen, Anhydrous Caffeine, Promethazine, Methylenedisalicylate |
| **Anticancer drugs** |  |  |
| Cyclophosphamide | Sorafenib | Leuprorelin |
| Tegafur, gimeracil, oteracil | Tegafur, Uracil | Calcium Folinate |
| Bicalutamide | Doxorubicin | Sunitinib |
| Crizotinib | Regorafenib | Gefitinib |
| Paclitaxel | Goserelin | Tamoxifen |
| Everolimus | Trastuzumab | Docetaxel |
| Temozolomide | Estramustine | Toremifene |
| Nilotinib | Nedaplatin | Epirubicin |
| Vincristine | Fluorouracil | Pazopanib |
| Abiraterone | Pembrolizumab | L-Asparaginase |
| Ipilimumab | Gemcitabine |  |
| **Gastrointestinal drugs** |  |  |
| Mosapride | Lansoprazole | Famotidine |
| Rebamipide | Esomeprazole | Rabeprazole |
| Cimetidine | Teprenone | Metoclopramide |
| Omeprazole | Ranitidine | Sofalcone |
| Polaprezinc | Troxipide | Azulene Sulfonate |
| Bifidobacterium | Clostridium butyricum MIYAIRI | Dried Yeast |
| Senna leaf | Sennoside A・B | Magnesium Oxide |
| Palonosetron | Itopride | Diastase |
| Salazosulfapyridine |  |  |
| **Psychiatric or neurological drugs** |  |  |
| Valproate | Carbamazepine | Etizolam |
| Mirtazapine | Lamotrigine | Risperidone |
| Donepezil | Piperidolate | Imipramine |
| Estazolam | Lithium carbonate | Fluvoxamine |
| Brotizolam | Phenytoin | Zolpidem |
| Zopiclone | Clomipramine | Trihexyphenidyl |
| Biperiden | Ramelteon | Memantine |
| Sulpiride | Clotiazepam | Chlorpromazine |
| Duloxetine | Loflazepate | Amitriptyline |
| Interferon beta-1a | Tizanidine |  |
| **Metabolic disease drugs** |  |  |
| Pravastatin | Atorvastatin | Allopurinol |
| Rosuvastatin | Glimepiride | Pitavastatin |
| Fluvastatin | Ezetimibe | Miglitol |
| Metformin | Omega-3-acidethyl esters | Fenofibrate |
| Voglibose | Epalrestat | Gliclazide |
| Pioglitazone | Sitagliptin | Liraglutide |
| Insulin human | Insulin Aspart | Insulin Glargine |
| Benzbromarone | Febuxostat |  |
